# Usability assessment in an E‐Health App for Cognitive Stimulation (LABPSI) in Elderly population

**DOI:** 10.1002/alz.092355

**Published:** 2025-01-09

**Authors:** Elsa Araceli Revollo Sarmiento, Gisela Noelia Revollo Sarmiento, Natalia Veronica Revollo Sarmiento, Maria Celeste López Moreno, Leticia Vivas

**Affiliations:** ^1^ IPSIBAT (UNMDP‐CONICET), Mar del Plata, Buenos Aires Argentina; ^2^ INECOA‐CONICET, San Salvador de Jujuy, Jujuy Argentina; ^3^ DCIC‐CONICET, Bahia Blanca, Buenos Aires Argentina; ^4^ IPSIBAT (UNMDP/CONICET), Mar del Plata, Buenos Aires Argentina; ^5^ IPSIBAT (CONICET/National University of Mar del Plata), Mar del Plata, Buenos Aires Argentina

## Abstract

**Background:**

Advances in the development of e‐health applications enhance the availability of preventive tools and the adherence to certain treatments in old age (Roberts et al., 2015; Granath et al., 2023). Using these apps provides benefits for cognitive stimulation, offering a technologically accessible option that promotes autonomy in the elderly. In this study, we present the app version of a web platform for cognitive stimulation in the elderly called LABPSI (https://labpsi.mdp.edu.ar). This lite version can be downloaded for android mobile phones and used offline. The app was developed based on user‐centered design, focusing on the elderly. The aim of this study is to assess the usability of the app version of LABPSI in this population.

**Methods:**

The sample consisted of 16 elderly people (81% women) with a mean age of 71.5 (DS = 8.72) and an average of 10.9 years of education.

Initially, users were introduced to the app providing the necessary time for use and exploration. Subsequently, they answered the MAUQ (Zhou L. et al., 2019) e‐health application usability questionnaire to assess participants' perceived ease of use, satisfaction, and utility when using LABPSI. The LABPSI final prototype was developed using App Inventor software (MIT APP INVENTOR, 2012).

**Results:**

The descriptive analysis of the MAUQ usability questionnaire variables were: a) ease of use; b) interface and satisfaction; c) utility. The first variable had a median value of 22 with a maximum questionnaire score of 25 and a range of responses of [6‐25]. The second variable had a median value of 30, with a maximum questionnaire score of 35, and a range of responses of [21, 35]. Finally, the utility variable scored a median of 26 with respect to the maximum questionnaire score of 30, with a range of [16‐30].

**Conclusions:**

This usability study on the new LABPSI cognitive stimulation app shows high levels of ease of use, satisfaction, and perceived utility by participants. The results demonstrate a good interaction between the elderly and the system, defining the app as a usable, appealing, and easy to learn software product for end‐users

